# The clinical features and initial pharmacotherapeutic options of children with Tic disorders

**DOI:** 10.3389/fped.2025.1636110

**Published:** 2025-09-17

**Authors:** Yuxin Xiang, Chang Tong, Dan Sun, Zhisheng Liu

**Affiliations:** Department of Neurology, Wuhan Children’s Hospital, Tongji Medical College, Huazhong University of Science and Technology, Wuhan, China

**Keywords:** Tic disorders, clinical manifestations, tic severity, pharmacotherapy initiation, pediatric patients

## Abstract

**Purpose:**

Tic disorders (TD) are common childhood neurodevelopmental conditions, characterized by diverse manifestations, leading to misdiagnosis and delayed therapy. Timely identification of TD and access to care can improve clinical outcomes. This retrospective study characterizes clinical features and initial pharmacotherapy in newly diagnosed pediatric TD.

**Method:**

This retrospective cohort study included 805 newly diagnosed pediatric TD patients. Tic severity was assessed using the Yale Global Tic Severity Scale (YGTSS), with patients stratified into mild (YGTSS scores < 25), moderate (25–50), and severe (>50) groups. Chi-square tests/Fisher-exact tests and Wilcoxon rank—sum tests compared group differences in baseline characteristics. Multivariate analyses identified factors associated with tic severity, and logistic regression analyses identified predictors of pharmacotherapy initiation.

**Results:**

In 805 subjects, 73.43%, 11.18% and 15.39% were classified into provisional tic disorder, chronic tic disorder, and Tourette syndrome (TS). The prevalence of comorbid attention-deficit/hyperactivity disorder (ADHD) was higher in moderate (21.45%) and severe (36.36%) groups than in the mild group (15.60%). The diagnosis of Tourette syndrome (aOR = 1.40, 95% CI: 1.23–160.31), age at onset (aOR = 1.63, 95% CI: 1.22–2.18), and age at diagnosis (aOR = 1.63, 95% CI: 1.22–2.17), comorbid ADHD (aOR = 7.12, 95% CI: 1.39–36.43) were positively associated with greater tic severity. Clonidine patch (CAP) and traditional Chinese medicine (TCM) were the most common choices initial pharmacotherapy in newly diagnosed pediatric TD. Scores of YGTSS, comorbid ADHD predicted treatment initiation.

**Conclusions:**

This study contributed insights into the clinical profiles across tic severity and pharmacotherapeutic approaches in newly diagnosed pediatric TD. The findings highlighted the independent associations between baseline factors and tic severity, as well as the predictors of pharmacotherapy initiation. CAP and TCM served as the most common choices in newly diagnosed pediatric TD.

## 1 Introduction

Tic disorders (TD) are childhood-onset neurodevelopmental conditions characterized by motor and/or phonic tics (1). Based on motor/phonic manifestations and courses of tics, TD are classified into three subtypes: provisional tic disorder (PTD), chronic tic disorder (CTD), and Tourette syndrome (TS). TD typically emerge around 5 years of age, peak in severity between 10 and 14 years and often tend to decline in adolescence (2). The severity of TD varies among individuals. In some cases, TD can impair daily functioning, affecting social interactions and academic performance (3). A survey shows that approximately 88% of patients report TD's negative influence on their daily lives (4). Severe or frequent tics may cause cervical spine injuries and neurological complications, including disc herniation, myelopathy, and even stroke due to traumatic vascular dissection (e.g., carotid/vertebral arteries) (5). Functional impairment generally worsens with increasing tic severity (6). Therefore, accurate diagnoses and timely intervention are essential to alleviate symptoms and reduce the overall disease burden in TD patients (7).

TD's clinical manifestations often overlap with other diseases, frequently resulting in misdiagnosis and delayed therapy (8). Indeed, prior studies have indicated a substantial diagnostic delay in TD, with intervals ranging from 3 to 12 years (8). Moreover, approximately 76%–90% of TD patients have comorbidities, including attention-deficit/hyperactivity disorder (ADHD), obsessive-compulsive disorder (OCD), oppositional defiant disorder (ODD), sleep problems, and anxiety and depression disorders (1, 9). The coexistence of comorbidities not only increases the risk of more severe and impairing symptoms but also considerably complicates the diagnostic process (1, 9).

The comprehensive management for TD comprise pharmacological, behavioral therapies and psychoeducation. Among these, pharmacological interventions have advantages in terms of their accessibility and convenience. Globally, aripiprazole, tiapride and clonidine are frequently recommended (1, 10, 11). In China, first-line medications for TD consist of antipsychotics, alpha agonists, traditional Chinese medicine (TCM) (11–13). ChangMaXiFeng Tablets contain traditional Chinese herbs derived from aqueous extracts of Gastrodia (Tianma) and Rhizoma Acori Tatarinowii (Cangpu); ShaoMaZhiJing Granules include Baishao (from Paeonia lactiflora) and Tianma; and JiuWeiXiFeng Granules comprise Gouteng (from Uncaria) and Tianma—all these TCM have been approved for TD therapy (14). In clinical practice, treatment decisions including whether to initiate pharmacotherapy and which agents to use greatly depend on clinicians' judgment and guardians' preferences (13). To date, evidence regarding optimal pharmacotherapy for newly diagnosed pediatric patients remains limited, particularly concerning how symptom severity and demographic factors (e.g., age, gender comorbidity) influence pharmacotherapeutic options.

Timely identification of TD and access to evidence—based care can improve clinical outcomes (15). However, existing studies on newly diagnosed pediatric TD populations remain scarce, particularly regarding nuanced clinical characteristics, real-world pharmacotherapy patterns, and the impact of demographic factors on treatment decisions. This retrospective study aims to describe the clinical characteristics and initial pharmacotherapy choices in pediatric patients newly diagnosed with TD, to compare features across different levels of tic severity, and to examine the association between demographic factors and both disease severity and treatment selection.

## 2 Methods and study design

This retrospective cohort study enrolled children aged 4–18 years who were newly diagnosed with TD at Wuhan Children's Hospital from October 2022 to October 2024, according to the Diagnostic and Statistical Manual of Mental Disorders, Fifth Edition (DSM-5) criteria. Tic severity was assessed using the Yale Global Tic Severity Scale (YGTSS), a well-established gold standard for evaluating TD. The severity of motor and phonic tics is assessed from aspects of their number, frequency, intensity, complexity and interference, as well as the impairment (16). YGTSS ratings were conducted by trained assessment physicians, all of whom received standardized training on this scale to ensure consistency and minimize subjectivity.

The inclusion criteria were as follows: (1) Diagnosed with TD according to DSM-5 criteria; (2) First visit to the clinic; (3) Aged 4–18 years; (4) Complete clinical data. Finally, a total of 805 patients were included in this study.

Demographic information included gender and age. The basic clinical information included age at symptom onset and diagnosis, clinical course (defined as the time interval from tic onset to diagnosis), TD subtypes, symptoms, comorbid ADHD, YGTSS scores, electroencephalogram (EEG) as well as medication options.

Patient data were retrieved from the scientific research data platform of Wuhan Children's Hospital, specifically from the hospital's specialized database for TD. This is a platform with preset safeguards to protect the patients' private information. The study was approved by the Medical Ethics Committee of Wuhan Children's Hospital (No. 2025R013-E01) according to *Measures for Ethical Review of Life Sciences and Medical Research Involving Humans in China*, and all patient privacy was strictly protected throughout the research process.

## 3 Statistical analysis

All data were analyzed using *R version 4.4.2*. Categorical variables were presented as frequencies and percentages. Normally distributed continuous variables were presented as mean ± standard deviation (SD), while skewed continuous variables were presented as median (25th, 75th percentiles).

Based on YGTSS scores, patients were classified into mild (<25), moderate (25–50), and severe tic groups (>50). Group differences in age at onset, clinical course, age at diagnosis, TD subtypes, tic symptoms, and premonitory tic were compared using continuity correction chi-square tests/Fisher-exact tests and Wilcoxon rank—sum tests.

Multivariate analyses were performed to identify factors associated with tic severity, with crude odds ratios (ORs) [95% confidence intervals (CIs)] and adjusted ORs (aORs) (95% CIs) reported. For the decision to initiate pharmacotherapy, logistic regression was applied to estimate ORs (95% CIs) and aORs (95% CIs) for initiating pharmacotherapy, providing an overview of clinical/demographic factors associated with the pharmacotherapeutic strategies. All *p*-values were two-tailed, and a *p* < 0.05 was considered statistically significant. For multiple pairwise comparisons between groups, *p*-values were adjusted using the Bonferroni correction to account for multiple testing. Additionally, we summarized the specific medications prescribed at initial diagnosis, including both monotherapy and combination therapy.

## 4 Results

### 4.1 Demographic and clinical features in pediatric TD patients

A total of 805 patients were included in the study, comprising 659 males and 146 females (male: female = 4.51:1). For TD subtypes, 73.43% (*n* = 591), 11.18% (*n* = 90), and 15.39% (*n* = 124) were classified into PTD, CTD, and TS, respectively. Among them, 144 cases (17.88%) had comorbid ADHD. The median age at diagnosis was 7.51 (6.07, 9.19) years and a median age at onset was 6.87 (5.24, 8.60) years. The median duration from symptom onset to diagnosis was 2.00 (1.00, 12.00) months.

Patients were classified into mild (*n* = 519, 64.47%), moderate (*n* = 275, 34.16%), and severe (*n* = 11, 1.37%) groups based on YGTSS. The five most common tic symptoms were eye blinking/eye rolling (*n* = 490, 60.87%), jaw/lip movement/spitting (*n* = 202, 25.09%), head movement (*n* = 196, 24.34%), throat clearing (*n* = 190, 23.60%), and grunting (*n* = 138, 17.14%). Twenty patients (2.48%) had a family history of TD in their first-degree relatives.

Among the patients, 27 (3.35%) exhibited EEG abnormalities. Of these, generalized epileptiform discharges were observed in 7 children, focal epileptiform discharges in 19 children, and diffuse slowing in 1 child. The anatomical locations of focal discharges were predominantly in the temporal (*n* = 9) and central (*n* = 9) regions.

### 4.2 Different clinical characteristics among different tic severity groups

No significant difference in gender distribution was observed across the mild, moderate, and severe tic severity groups. The prevalence of PTD diagnosis differed significantly among the groups, with a higher proportion in the mild tic group (74.95%) than in the moderate (70.54%) and severe (72.27%) groups (*χ*^2^ = 21.61, *p* < 0.01, Cramer's V = 0.12) (Table 1). The prevalence of comorbid ADHD was higher in moderate (21.45%) and severe (36.36%) groups than in the mild group (15.60%) (*χ*^2^ = 6.78, *p* = 0.03, Cramer's V = 0.09). The median duration from tic onset to diagnosis was 2.00 (0.66, 12.00), 3.00 (1.00, 12.00) and 5.00 (1.66, 9.08) months in mild, moderate, and severe TD groups, respectively (pairwise comparison *p* < 0.01 for mild vs. moderate).

**Table 1:** Clinical features of pediatric patients newly diagnosed with tic disorders among the 3 groups.

Median (25th, 75th)/*N* (%)	Mild tic group (*n* = 519)	Moderate tic group (*n* = 275)	Severe tic group (*n* = 11)	*p* value
Male^a^	417 (80.35)	232 (84.36)	10 (90.90)	0.28
Female	102 (19.65)	43 (15.64)	1 (9.10)	
Age at onset (year)^b^	6.94 (5.39, 8.61)	6.81 (5.39, 8.49)	8.04 (5.94, 9.51)	0.35
Clinical course (month)^b^	2.00 (0.66, 12.00)	3.00 (1.00, 12.00)	5.00 (1.66, 9.08)	<0.01
Age at diagnosis (year)^b^	7.50 (6.00, 9.05)	7.48 (6.18, 9.54)	8.57 (6.97, 10.22)	0.14
ADHD^a^	81 (15.60)	59 (21.45)	4 (36.36)	0.03
Provisional tic disorder^a^	389 (74.95)	194 (70.54)	8 (72.27)	<0.01
Chronic tic disorder	70 (13.48)	19 (6.90)	1 (9.09)	
Tourette syndrome	60 (11.56)	62 (22.54)	2 (18.18)	

^a^ Continuity Correction Chi—Square Test.

^b^ Wilcoxon rank-sum test.

ADHD, attention-deficit/hyperactivity disorder.

The prevalence of vocal tics was significantly higher in the moderate TD group (throat clearing: 33.45%; grunting: 25.09%) than in the mild TD group (throat clearing: 18.49%; grunting: 12.52%) (pairwise comparison *p* < 0.01) (Table 2).

**Table 2:** Symptoms of pediatric patients newly diagnosed with tic disorders among the 3 groups.

*N* (%)	Mild tic group (*n* = 519)	Moderate tic group (*n* = 275)	Severe tic group (*n* = 11)	*p* value
Eye blinking/eye rolling	319 (61.46)	168 (61.09)	3 (27.27)	0.07
Jaw/lip movement/spitting	130 (25.04)	69 (25.09)	3 (27.27)	0.98
Head movement	125 (24.08)	69 (25.09)	2 (18.18)	0.84
Throat clearing	96 (18.49)	92 (33.45)	2 (18.18)	<0.05
Grunting	65 (12.52)	69 (25.09)	4 (36.36)	<0.05

Multivariate analyses revealed that the diagnosis of Tourette syndrome (aOR = 1.40, 95% CI: 1.23–160.31), age at onset (aOR = 1.63, 95% CI: 1.22–2.18), and age at diagnosis (aOR = 1.63, 95% CI: 1.22–2.17) were positively associated with greater tic severity. The prevalence of comorbid ADHD demonstrated a positive association with higher tic severity (aOR = 7.12, 95% CI: 1.39–36.43) (Table 3).

**Table 3:** Multivariate analyses of the association between demographic factors and tic severity.

Demographic factors	OR (95% CI)	aOR (95% CI)
Male^a,^*	4.5 (0.86–15.61)	2.49 (0.46–12.55)
Clinical course^b^	1.08 (1.02–1.14)	1.00 (0.98–1.01)
Chronic tic disorder^c,^**	0.14 (0.09–1.09)	0.03 (0.01–0.41)
Tourette syndrome^c,^**	72.96 (12.26–421.26)	1.40 (1.23–160.31)
Attention-deficit/hyperactivity disorder^d^	12.16 (2.32–63.49)	7.12 (1.39–36.43)
Age at onset^e^	1.47 (1.11–1.96)	1.63 (1.22–2.18)
Age at diagnosis^e^	1.76 (1.33–2.32)	1.63 (1.22–2.17)
Premonitory urge^f^	0.16 (0.01–2.79)	0.12 (0.01–2.09)

^a^ Adjusted clinical course, diagnoses, ADHD (attention-deficit/hyperactivity disorder), age at diagnosis, premonitory urge.

^b^ Adjusted gender, diagnoses, ADHD, age at diagnosis, premonitory urge.

^c^ Adjusted gender, clinical course, ADHD, age at diagnosis, premonitory urge.

^d^ Adjusted gender, clinical course, diagnoses, age at diagnosis, premonitory urge.

^e^ Adjusted gender, clinical course, ADHD, diagnoses, premonitory urge.

^f^ Adjusted gender, clinical course, diagnoses, ADHD, age at diagnosis.

* The reference group is the female group.

** The reference group is the provisional tic disorder group.

### 4.3 The initial pharmacotherapy options of TD

**Figure 1:**
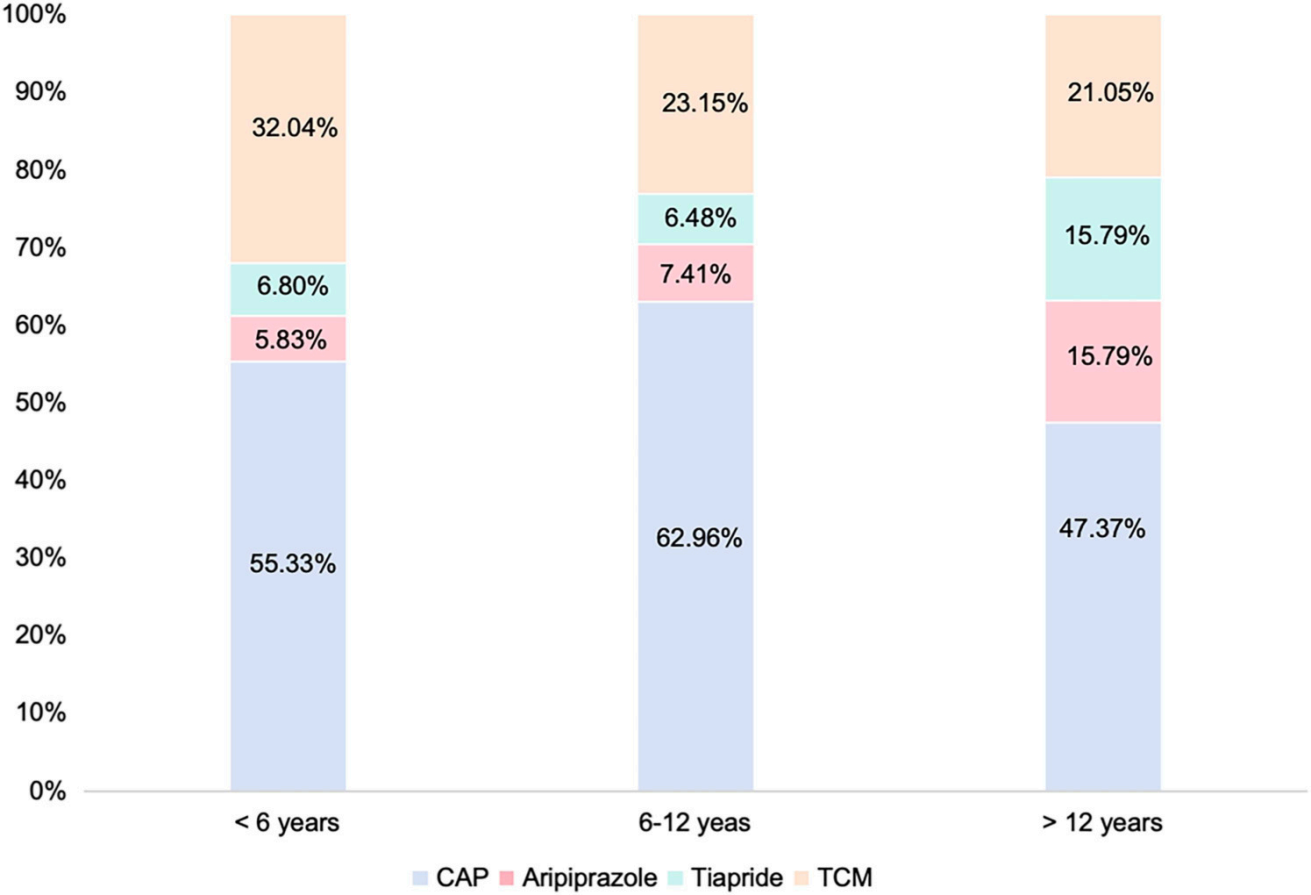
In the monotherapy group, the proportion each medication of age groups. CAP, clonidine adhesive patches; TCM, traditional Chinese medicine.

Pharmacotherapy was initiated in 75.4% (*n* = 607) of patients at initial TD diagnosis. Among these, 161 (26.52%) received combination therapy, and 446 (73.48%) adopted monotherapy. Within the monotherapy group, clonidine adhesive patch (CAP) constituted the primary choice (*n* = 270, 60.53%), followed by TCM (*n* = 112, 25.11%) (Table 4). Within prespecified age subgroups (<6 years, 6–12 years, >12 years; Figure 1), TCM and CAP remained the most common options (though *p* = 0.21). In the combination therapy group, the majority were prescribed CAP + TCM (*n* = 83, 51.56%), followed by CAP combined with antipsychotics (*n* = 43, 26.71%).

**Table 4:** Pharmacotherapy choices for pediatric patients newly diagnosed with tic disorders.

Monotherapy group (*n* = 446)	Frequency (N)	Percentage (%)
CAP	270	60.53
TCM	112	25.11
Aripiprazole	33	7.40
Tiapride	31	6.96
The combined therapy group (*n* = 161)		
CAP + TCM	83	51.56
CAP + antipsychotic	43	26.71
TCM + antipsychotic	14	8.70
Others	21	13.03

CAP, clonidine adhesive patch; TCM, traditional Chinese medicine.

Logistic regression analysis was used to identify the potential factors associated with initiating pharmacological intervention at initial TD diagnosis. The YGTSS score was associated with starting pharmacological treatment (total score: aOR = 1.06, 95% CI: 1.04–1.08; motor score: aOR = 1.04, 95% CI: 1.01–1.09; vocal score: aOR = 1.09, 95% CI: 1.05–1.13; impair score: aOR = 1.04, 95% CI: 1.01–1.08). The comorbid ADHD was related with the odds of starting pharmacotherapy (aOR = 1.82, 95% CI: 1.09–3.04) (Table 5).

**Table 5:** The association between demographic factors and the option of starting pharmacological treatment.

Demographic factors	OR (95% CI)	aOR (95% CI)
Total score of YGTSS^a^	1.07 (1.04–1.09)	1.06 (1.04–1.08)
Motor score of YGTSS^a^	1.04 (1.00–1.09)	1.04 (1.01–1.09)
Vocal score of YGTSS^a^	1.10 (1.06–1.14)	1.09 (1.05–1.13)
Impair score of YGTSS^a^	1.05 (1.02–1.09)	1.04 (1.01–1.08)
Clinical course^b^	1.02 (1.01–1.04)	1.00 (0.99–1.01)
Age at diagnosis^c^	1.12 (1.04–1.21)	1.06 (0.98–1.15)
Chronic tic disorder^d,^*	1.12 (0.67–1.87)	0.89 (0.44–1.79)
Tourette syndrome^d,^*	3.30 (1.81–6.03)	1.87 (0.84–4.17)
Male^e,^**	1.07 (0.71–1.61)	0.90 (0.59–1.39)
Attention-deficit/hyperactivity disorder^f^	2.19 (1.33–3.59)	1.82 (1.09–3.04)

^a^ Adjusted clinical course, age at diagnosis, diagnoses, gender, ADHD (attention-deficit/hyperactivity disorder).

^b^ Adjusted total score of YGTSS, age at diagnosis, diagnoses, gender, ADHD.

^c^ Adjusted total score of YGTSS, clinical course, diagnoses, gender, ADHD.

^d^ Adjusted total score of YGTSS, clinical course, age at diagnosis, gender, ADHD.

^e^ Adjusted total score of YGTSS, clinical course, age of diagnosis, diagnoses.

ADHD.

^f^ Adjusted total score of YGTSS, clinical course, age at diagnosis, diagnoses, gender.

* The reference group is the provisional tic disorder group.

** The reference group is the female group.

YGTSS, Yale Global Tic Severity Scale.

Tic severity stratified analysis results showed a clear gradient: pharmacological treatment was used in 100% of severe, 86.9% of moderate, and 68.2% of mild tic severity group cases (*χ*^2^ = 37.26, *p* < 0.01, Cramer's V = 0.215). Combination therapy was more prevalent in moderate (32.21%) and severe (45.45%) groups compared with mild (21.46%) (*χ*^2^ = 10.69, *p* < 0.01, Cramer's V = 0.13).

## 5 Discussion

TD are common neurodevelopmental conditions with marked variability. It is estimated that approximately 73% of patients are initially misdiagnosed, underscoring the importance of early and accurate diagnosis for initiating timely interventions (7, 8). TD management requires personalized strategies, with decisions based on guardians' preferences, comorbidities, age, tic severity, clinician judgment, expertise, and regional guidelines (17). However, relevant evidence remains limited.

Clinically, due to subtle or atypical early symptoms, many pediatric patients initially consult other departments such as ophthalmology or otolaryngology—before going to neurology. Prior studies have shown that tic symptoms often originate in the face and head (8, 14). According to Park's research (involving 117 pediatric patients), the most common symptoms in TD are eye blinking (50.40%) followed by jaw/lip movement (29.40%) and throat clearing (29.40%) (8). Similarly, in Nilles et al' study (involving 203 pediatric and adult patients), the most common symptoms are eye blinking (57%), head tics (51%), eye rolling (48%), mouth movements (46%), and throat clearing (42%) (18). Another study shows that ocular tics occur in over 90% of individuals with TS (19). Consistent with these reports, our research also find eye blinking/eye rolling to be the most common symptoms (60.87%). These findings suggest that although the specific presentation of TD may vary across populations, motor tics predominantly affect the head and face, with eye-related movements being most common (typically >50%), while throat clearing represents a frequent vocal tic. That is the reason why many patients first seek medical treatment at the ophthalmology or otolaryngology department. Therefore, in children presenting with chief complaints like repetitive eye blinking or throat clearing, clinicians should consider the possibility of TD, and evaluate for additional tic-related symptoms to reduce the risk of misdiagnosis or delayed diagnosis.

 TD patients frequently present with comorbid psychiatric conditions, significantly impairing quality of patient life. ADHD is the most prevalent comorbidity, affecting approximately 20% of TD patients and 50%–60% of those with TS (20, 21). In our study, comorbid ADHD is more prevalent in severe (36.60%) and moderate (21.45%) TD groups compared to the mild group (15.60%). Additionally, ADHD is associated with increased tic severity (aOR = 7.12, 95%CI: 1.39–36.43). In fact, compared with TD patients without ADHD, patients with TD and co-occurring ADHD also have greater functional impairment (22). And Cols et al. find that ADHD is a strong risk factor for tic persistence (OR = 3.35, 95% CI: 2.82–3.99) (23). Clinically, these comorbidities not only complicate the diagnosis of TD but also contribute to impairments in academic performance and social functioning, thereby diminishing quality of life for both patients and their families. Notably, in our study, the comorbid ADHD is even seemingly related with the odds of initiating pharmacotherapy of TD (aOR = 1.82, 95% CI: 1.09–3.04). Given the substantial impact of ADHD comorbidity, clinicians may consider addressing ADHD alongside TD in such patients. In Japan, most clinicians indicate atomoxetine as the first-line medication for comorbid ADHD and extended-release guanfacine has been approved for tics in children with ADHD (10). US guidelines suggest that clonidine, methylphenidate, guanfacine, and combination therapies (e.g., clonidine plus methylphenidate) may effectively reduce both tic severity and ADHD symptoms (11).

EEG is a sensitive tool for monitoring abnormal brain activity and plays a vital role in the diagnosis of paroxysmal neurological conditions. It is also well-suited for assessing cerebral function in children with TD (24). In our study, abnormal EEG findings—predominantly epileptiform discharges—were observed in 27 patients (3.35%), though these discharges did not occur synchronously with tic episodes. Some researchers propose that such abnormalities may reflect underlying neural dysfunction and disrupted brain network activity (25). So, this raises an important question: could the presence of non-specific epileptiform discharges indicate functional impairments in cortical regions responsible for suppressing involuntary motor and vocal tics? However, relevant research supporting this hypothesis is currently limited. In the future, investigations can be conducted into the mechanism of non-specific discharges in children with TD, and even combined with the prognosis of TD.

Pharmacological intervention remains the most common approach for tic therapy. However, the decision to initiate treatment and selection of specific modalities rely on clinicians' expertise and caregiver preferences. Meanwhile, international clinical recommendations for pharmacotherapy of TD vary slightly across regions. A Japanese consensus suggests aripiprazole as the first-line medication and risperidone as the second-line option for TD (10). A survey of Canadian physicians indicates that aripiprazole, risperidone, and clonidine are the most frequently prescribed medications for managing TD (26). In contrast, European guidelines recommend aripiprazole as the first-line treatment, with risperidone and tiapride designated as second-line medications (17). Wang et al. conduct a study showing that for patients under 6 years old, TCM and CAP are the most commonly prescribed medications, and the utilization of antipsychotics presents an upward trend as age increased (11). In our study, TCM and CAP are the most common choices across all age groups. This discrepancy may be attributed to differences in study populations: Wang et al.'s cohort included both newly diagnosed and previously treated children—some of whom may have switched to antipsychotics after CAP/TCM failed (11). Moreover, in this study, the sample sizes of the age groups under 6 years old and 12 years old and above are relatively small. Therefore, these prescribing trends may not fully reflect broader real-world patterns.

Notably, antipsychotics such as aripiprazole and tiapride—internationally proven as first-line treatments for TD—have a low usage rate among newly diagnosed pediatric TD patients in this study. This may be attributed to the fact that all participants in the study were newly diagnosed. In China, many parents may be reluctant to use oral Western medicine, especially in the early stages of the disease, when they tend to try TCM instead. Additionally, the package insert for aripiprazole tablets in China does not list TD as an indication (only schizophrenia is included), which may make it hard for some families to accept.

Both alpha2-adrenergic receptor agonists, antipsychotics and TCM have been approved for TD therapy for their efficacy and safety. However, questions still remain to be answered: when to initiate pharmacotherapy and how to select specific agents? A meta-analysis points that the preference of clinicians and caregivers impact medicine prescription (27). Prospective studies are limited due to TD's long-term course. While our study identifies baseline factors associated with TD severity (e.g., ADHD comorbidity, tic duration), Ricketts et al. further showed that gender and childhood tic severity predict adult tic severity (28). Future research should explore additional modifiable risk factors to refine personalized treatment algorithms.

This study has several limitations that should be considered. First, as a single-center retrospective study, the generalizability of the findings may be limited, particularly to populations in different geographic regions or health care systems with distinct diagnostic and treatment protocols. Furthermore, this observation is only applicable to the specific context of clinical practice in China, and we should clarify that there are significant differences in treatment options and regulatory environments across different regions. Additionally, due to the small sample size in some subgroups, formal sensitivity analysis was not feasible; thus, we were unable to control for potential selection or prescription bias in these comparisons, which merits attention. Second, the small number of severe TD cases (*n* = 11) may introduce bias and reduce the reliability of findings within this subgroup. Third, although TD is commonly associated with various comorbidities such as OCD and anxiety/depressive disorders, this study focused solely on ADHD, which limits further exploration of the comprehensive relationship between TD and their comorbidities. These limitations highlight the need for multicenter, prospective studies with larger sample sizes and even longer follow-up periods to corroborate our findings and enhance the generalizability of the results.

## 6 Conclusions

In summary, this study contributes insights into the clinical profiles across tic severity and pharmacotherapeutic approaches in newly diagnosed pediatric TD. We emphasize the independent associations between baseline factors and tic severity, as well as the predictors of pharmacotherapy initiation. CAP and TCM serve as the most common choices in newly diagnosed pediatric TD.

## Data Availability

The data analyzed in this study is subject to the following licenses/restrictions: The datasets generated during and/or analysed during the current study are available from the corresponding author on reasonable request at liuzhisheng@hust.edu.cn. Requests to access these datasets should be directed to liuzhisheng@hust.edu.cn.
